# Investigation of natural radionuclides and radiation shielding potential of some commonly used building materials in Northwestern Nigeria

**DOI:** 10.1038/s41598-024-60541-5

**Published:** 2024-04-27

**Authors:** N. N. Garba, A. S. Aliyu, N. Rabiu, U. M. Kankara, A. M. Vatsa, A. Ismaila, J. Musa, E. Onuh

**Affiliations:** Department of Physics, Ahmadu Bello University, Zaria, Nigeria

**Keywords:** Building materials, Gamma ray, Shielding, Py-MLBUF, Nigeria, Environmental sciences, Physics

## Abstract

This study assessed the gamma-ray shielding potential of clay, sand, gypsum and kaolin commonly used as a building material in Northwestern, Nigeria. The radiological status of the samples was first evaluated by determining the activity concentrations of ^238^U, ^232^Th and ^40^K using NaI (Tl) detector after which elemental composition and mass density were determined using Neutron Activation Analytical Technique. After which the mass attenuation coefficients (MAC), linear attenuation coefficients (LAC), half value layer (HVL), tenth value layer (TVL), and effective atomic number (Zeff) were determined experimentally and theoretically using standard point sources of ^137^Cs and ^60^Co photon peaks (0.662 and 1.332, 1.173) MeV and Py-MLBUF software. The activity concentrations were found to range from 51 to 59 Bq kg^−1^ with a mean value of 59 Bq kg^−1^ for ^238^U, 24 to 27 Bq kg^−1^ with a mean value of 29 Bq kg^−1^ for ^232^Th, and 219 to 247 Bq kg^−1^ with a mean value of 247 Bq kg^−1^ for ^40^K which were about within the world recommended values of 33, 45 and 420 Bq kg^−1^ respectively. The results of the elemental compositions show that Si, Al, K, Fe, and Ba in clay and sand samples have concentrations in the range of 36.83–78.48%, 1.92–26.05%, 6.33–21.96%, 2.39–19.09%, and 0.09–1.44%, respectively, while in kaolin and gypsum, results revealed that Si, Al, K, Fe, and Ca range between 0.34 and 65.52%, 1.14–35.82%, 0.00–12.12%, 0.00–5.77%, and 0.00–96.6%, respectively. However, the concentrations of other elements such as Mg, Ti, Mn, Zn, Na, and Ba varied significantly with the samples. The results showed that clay has an average density of 1.96 g/cm^3^, sand has 2.32 g/cm^3^, kaolin has 2.63 g/cm^3^, and gypsum has the highest density with a value of 2.66 g/cm^3^ compared to other samples. During the measurements, a thallium-activated sodium-iodide NaI (TI) detector was used. A narrow beam transmission geometry condition was adopted for the measurements to ensure minimal scattered radiation. Absorption and attenuation of gamma beams as a function of sample thickness against gamma energy generally exhibit an increasing gamma ray behaviour as the sample thickness increases from 1 to 3 cm. The results showed that the gypsum, kaolin, sand, and clay were capable of attenuating 63.5%, 61.5%, 58.4%, and 44.2 of gamma-ray photons of energy 0.662 MeV at 3 cm thickness %, respectively, and 40.6%, 32.9%, 30.6%, and 27.3% of gamma energy 1.332 MeV at 3 cm thickness, respectively. The results showed that MAC, LAC, and Zeff of all the samples decreased with an increase in photon energy, while those of HVL and TVL increased. The experimental results for all the gamma-ray shielding parameters were found to be in good agreement with the theoretical values obtained using Py-MLBUF. The results have shown that all the samples have similar photon attenuation behaviours; however, gypsum has the best shielding potential than kaolin and this is attributed due to its highest density value.

## Introduction

The application of radiation sources in various fields and the use of gamma ray-emitting isotopes in nuclear research, industry, medicine, agriculture and education in the world was increasing^1^. When radiation interacts with material, it loses some of its energy by deposition into the atoms of the matter. Ionizing radiation such as gamma-radiation sources that are used in nuclear medicine, contribute to improve people’s quality of life, it also represents a risk^2^. Ionizing radiation causes the ionization of the cell's atoms, thereby destroying the normal chemical equilibrium of the cell and eventually can lead to the cell's death or permanent deformation^3^.

Terrestrial radionuclides that can be found on Earth typically emerged during the formation of the planet^4,5^ and some of them takes several years to decay and cease to be radioactive, but they remain a part of the system^4,5^. They are known to originate in the earth and enter the environment through soil, air, water, and building materials^5,6^. It stands to reason those natural radionuclides such as ^238^U, ^232^Th and ^40^K are contains in these materials at different proportions, and are used for different purposes radiation shielding inclusive^5,7,8^.

Since radiation can pose a serious concern in medical X-ray systems, industries, nuclear reactors and many other instances, then radiation shielding is imperative, in order to protect the general public and workers involved in radiation related fields from the radiation effects and patients exposed to ionizing radiation for treatment purposes^9^. Containing radiation and preventing it from causing physical harm to patients, employees, and or their environment is an integral part of operating equipment that emits radiation. Preserving human and structural material that may be compromised from radiation exposure is very necessary with different radiation-shielding materials better suited than others as determined by the interaction between radiation and the elemental properties of the shielding materials^5,10^.

Radiation shielding is based on the principle of attenuation, which is the ability to reduce the dose by completely absorbed or scattered when passed through a material medium. When radiation interacts with a material it loses some of its energy through different kinds of interactions such as photo-electric effect, Compton’s effect and pair production^3^. Radiation shielding, which involves using materials to reduce radiation intensity and protect against radiation damage, is essential in various fields. For decades, lead has been considered as the most often use in radiation shielding. It is commonly used for X-rays and gamma radiation shields because of its high density, high atomic number and therefore with high linear attenuation coefficients^11^. It is easy to process and provides durable shielding, but yet there has been an overwhelming increase in health, safety, and environmental concerns over the mining, processing, handling, and its disposal^5^. Lead can be hazardous when taken into the body by swallowing or breathing in lead or materials contaminated with lead. In fact, lead has already been banned from use in many applications, such as motor fuels, paint, and water pipes in many countries and its total prohibition in several countries^12^.

Recognizing the need for an alternative shielding material, researchers have sought for materials that are nontoxic, environmentally friendly, and capable of providing reliable radiation protection^13–15^. For decades, local building materials, such as clay, gypsum, kaolin, and sand were used as raw materials for building in the world, research have shown that these materials can be used for the purpose of radiation shielding in order to protect the general public and the personnel from the harmful effects of radiation. They were found to be promising candidates and offer potential substitutes for lead due to their high density. These materials are abundant in various quantities across the study area. Therefore, there is always a need to develop new and improved materials that can be used under potential harsh conditions of radiation exposure and act as shielding materials for extended periods of time^15–17^.

For the selection of an appropriate material for gamma ray shielding, shield-designers have to investigate materials’ suitability by using its Gamma-ray Shielding Parameters (GSP) (GSP)^10,18^. Garba et al., in 2023, assessed the radiological status of some commonly used building materials (clay, sand, kaolin and gypsum) in Northwestern Nigeria and suggested that they can further be explored for radiation shielding application^5^.

Therefore, this study investigates the gamma radiation shielding behaviours of clay, sand, kaolin and gypsum commonly used as building materials in Northwestern Nigeria. For this reason, several parameters such as attenuation effectiveness, HVL, TVL and Z_eff_ were determined.

## Materials and methods

Four different locally used building materials (clay, sand, kaolin, and gypsum) from the areas of (Zamfara, Sokoto, Kebbi, Katsina, Kano, Jigawa and Kaduna) in Northwestern Nigeria were obtained with the aid of local suppliers. Then prepared for analysis to ascertain their experimental and theoretical gamma ray shielding behaviours using standard point sources of Cesium-137 and Cobalt-60 of photon peaks (0.662 and 1.133, 1.173) MeV and Py-MLBUF software. During the measurements, thallium activated sodium iodide NaI(TI) detector was used. The elemental compositions of the samples were determined using Neutron Activation Analysis (NAA) at the Centre for Energy Research and Training (CERT) Ahmadu Bello University, Nigeria. The choice of NAA technique was because it is fast, accurate and non-destructive technique.

### Sample collection and preparation

The selected materials were collected in plastic polyethylene bags, weighed, packed, and transported to the laboratories at the Physics Department and Centre for Energy Research and Training, Ahmadu Bello University, Zaria for preparation and analysis. The collected samples were oven dried at 105 °C for 24 h in order to remove moisture contents and then allowed to cool down to room temperature and later crushed, grounded and then sieved for homogeneity using 500 μm and 250 μm mesh^5,19^.

### Methodology

#### Measurement of Activity Concentrations of ^238^U, ^232^Th, and ^40^K

The prepared samples were analysed for the activity concentrations of ^238^U, ^232^Th, and ^40^K using NaI (Tl) gamma ray detector situated at the Centre for Energy Research and Training, Ahmadu Bello University, Zaria. The detector consists of a 7.62 × 7.62 cm NaI (TI) detector housed in a 10 cm thick lead-shield, cadmium-lined assembly with copper sheets for the reduction of background radiation, which was equipped with a MAESTRO computer system program for data acquisition and spectra analysis. IAEA standard reference materials (RGK-1, IAEA-448, and RGTh-1) were used for the quantitative analysis of the ^238^U, ^232^Th, and ^40^K, respectively^5,20^. Each of the prepared samples was counted for approximately 4 h, with the activity concentration of ^238^U determined using the gamma energy line of ^214^Bi (1760 keV), ^232^Th determined using ^208^Tl (2614 keV), while the activity concentration of ^40^K was determined directly from its 1460 keV gamma line. Energy and efficiency calibrations were carried out using a point source of 500 mL of ^60^Co (1173 and 1332 keV), ^241^Am (59.54 keV), and ^137^Cs (661.62 keV) multi-nuclide standard solution, respectively. The net number of counts under each photo peak of interest was then background subtracted using the time-correct spectrum taken using the blank container and the specific activity concentrations of the radionuclides were calculated using Eq. (1)^5,21^.

1 $${\text{A}}=\frac{{\text{N}}-{{\text{N}}}_{{\text{o}}}}{{{\text{I}}}_{\upgamma }\mathrm{\varepsilon mt}},$$

where A is the specific activity of the radionuclide in the sample, N is the net counts of a given peak for a sample, N_o_ is the background count of the given peak, Iγ is the number of gamma photon per disintegrations, ε is the detector efficiency at the specific gamma-ray energy, m is the measured mass of the sample, and t is the measuring time^5^

#### Measurement of elemental compositions using neutron activation analysis (NAA)

The samples analysed using Nigerian Research Reactor-1 (NIRR-1) at the Centre for Energy Research and Training, Ahmadu Bello University. NIRR-1 is a low-power nuclear reactor, which has highly enriched uranium as fuel, light water as moderator and beryllium as reflector^22^. The prepared samples were exposed to neutrons causing a portion of the atoms to undergo neutron capture, which produces high-energy compound nuclei that rapidly transform into radioactive forms of the original chemical element(s). As the radioactive isotopes undergo decay to reach stable ground state configurations, the sample is placed on a high-purity germanium detector, which records the intensities and energies of the gamma rays that are emitted^23,24^. The radioactive emissions and their decay paths for each element are well known. The radioactive isotope always emits gamma rays at certain specific energies and intensities. The radioisotopes present, and hence the parent chemical element(s) present in the sample, was determined quantitatively specified by comparison with the activities of the standard reference materials^23,25^.

#### Measurement of gamma ray shielding parameters (GSP)

Information about various gamma-ray shielding parameters (GSP) is required to investigate the gamma-ray shielding efficacy of the materials. The effectiveness of a shield depends on the energy of the photons and on the type of the shielding material. The higher the atomic number and the density of the shielding material the more effective it is in reducing the intensity of gamma radiations.

##### Theoretical assessment of GSP

For the simulation of gamma ray shielding parameters of the studied samples, Python-program for Multi-Layered Build-Up Factors (Py-MLBUF) computer code was used in computing the gamma ray shielding parameters in the photon energy range of (0.1–5.0) MeV. In achieving these, the samples elemental compositions (by weight) and densities were used as input parameters to the computer code^18^.

##### Experimental assessment of GSP

The samples were prepared by mixed with distilled water, and then a homogeneous mixture of each sample was obtained and cast into blocks of 1, 2, and 3 cm thicknesses for each of the samples. After 24 h, the blocks were removed from the moulds and placed in a plastic container. Special care was taken in order to preserve their physical intactness when carrying them to the laboratory for the experiment.

Furthermore, the mass density of the prepared samples was determined from the calculated mass of each sample using a digital beam balance, and the volume of each sample was calculated from the dimensions of the moulded blocks and the density was obtained using Eq. (2).

2 $$ \rho = \frac{m}{V}\left( {{\text{kg}}/{\text{m}}^{{3}} } \right), $$

where, m and V are the mass and volume of the sample respectively.

**Figure 1:**
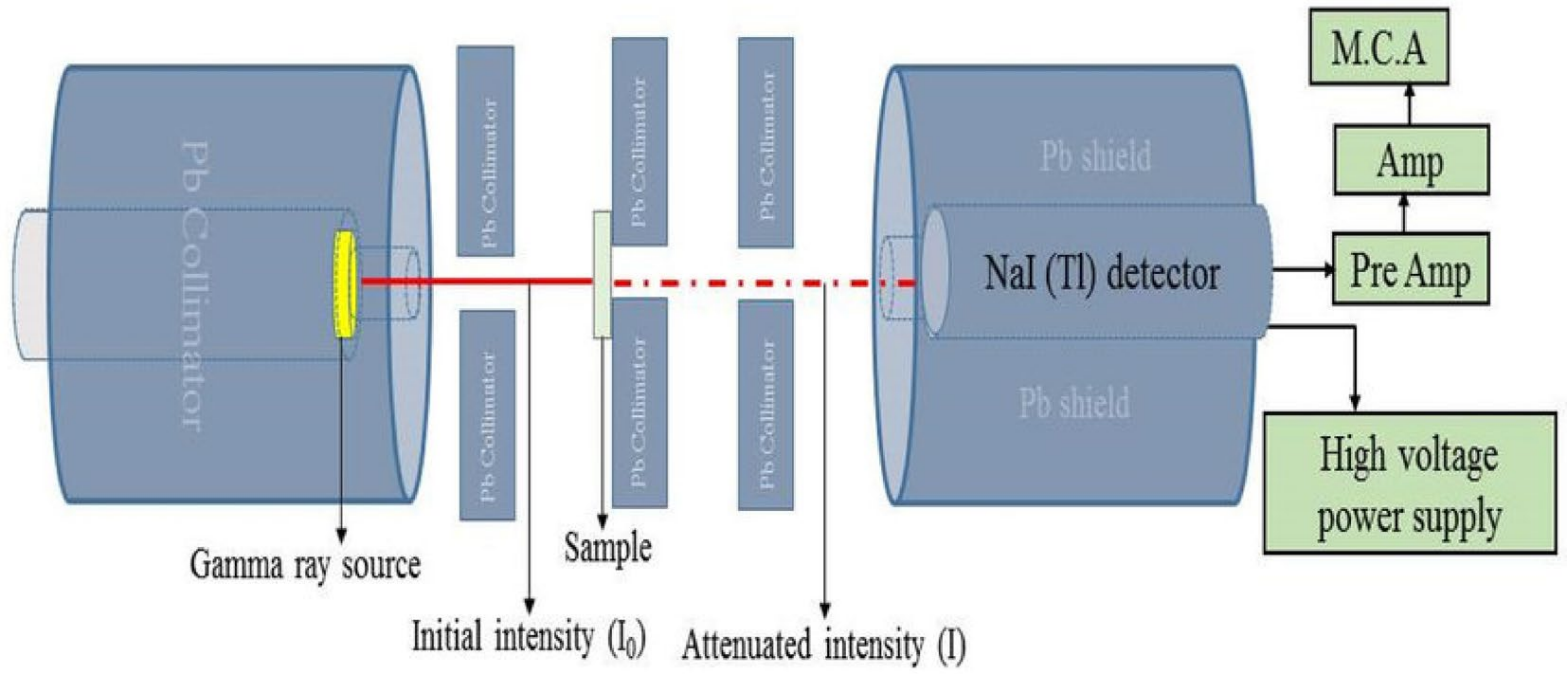
Experimental set-up of the narrow beam transmission geometry^26^.

The gamma ray shielding measurements of the prepared samples were performed using NaI (TI) detector of dimension 7.62 × 7.62 cm housed in a 10 cm thick lead-shield at Centre for Energy Research and Training, Ahmadu Bello University Zaria, Nigeria. Cesium-137 and Cobalt-60 gamma ray point sources of photon peaks (0.662 and 1.133, 1.173) MeV were used. The incident and transmitted gamma-ray intensities of the point sources due to the presence of the samples were determined for the measurements of mass attenuation coefficients. A narrow beam transmission geometry condition was adopted for the measurements to ensure minimal scattered radiation as shown in Fig. 1.

The total mass attenuation coefficient (MAC), linear attenuation coefficient (LAC), half-value layer (HVL), tenth value layer (TVL) and effective atomic number (Z_eff_) for the gamma ray shielding of the samples were evaluated using Eqs. (3–6) respectively.

3 $${\text{MAC}}=\sum {{\text{w}}}_{{\text{i}}}{\left({\text{MAC}}\right)}_{{\text{i}}},$$

where, ${w}_{i}$ is the weight fraction, (MAC) represent the mass attenuation coefficient of the ith constituent^18^. The linear attenuation coefficients (LACs) which indicates the rate at which photons interact as they move through material and is inversely related to the average distance photons travel before interacting are calculated Eq. (4)^27^.

4 $${\text{LAC}}= \frac{-{\text{In}}(\frac{{\text{I}}}{{{\text{I}}}_{{\text{o}}}})}{{\text{x}}}. $$

The half-value layer (HVL) of the absorbing material which is a measure of the thickness that decrease the incident photons intensity to half of its initial value is calculated using Eq. (5)^27,28^.

5 $$\mathrm{HVL }= \frac{{\text{ln}}2}{{\text{LAC}}}.$$

The tenth-value layer of the absorbing material which represents the thickness that decrease the incident photons intensity to one tenth of its initial value was calculated using Eq. (6)^28,29^.

6 $$\mathrm{TVL }= \frac{{\text{ln}}10}{{\text{LAC}}}.$$

Additionally, the effective atomic number (Z_eff_) which is an important parameter in radiation shielding that describes the attenuation of gamma and X-ray photons radiations for any material with high values of it indicating better attenuation of gamma and X-ray photons was calculated using Eq. (7)^30^.

7 $$ Z_{eff} = \frac{{\sigma_{a} }}{{\sigma_{e} }}, $$

where σ_a_ and σ_e_ are the total atomic and electric cross-section of the materials^18,29^.

## Results and discussion

### Activity concentrations of ^226^Ra, ^232^Th and ^40^K and elemental composition

The activity concentrations of ^238^U, ^232^Th and ^40^K, the elemental compositions and densities of the samples determined were presented in Tables 1 and 2 respectively. Generally, it can be observed from Table 1 that, the activity concentration of ^40^K was found to be the highest in all the analysed samples of the four commonly used building materials within the study area, then followed by ^2226^Ra with ^232^Th having the lowest. This clearly indicates that ^226^Ra and ^40^K are the major sources of gamma radiation in the studied samples and this is in good agreement with a similar studies conducted in Kerala, India^31^. Additionally, the relatively high amount of ^40^K observed in all the samples though not above the world average value, can be attributed to the amount of potassium silicate in the geological formations of area and the intense agricultural activities in the environment^5^. Therefore, it can be concluded that the samples are radiologically free and thus have the potential to be utilized for radiation shielding purposes.

**Table 1:** Activity concentrations of ^238^U, ^232^Th and ^40^K.

Sample code	Activity concentration (Bq kg^−1^)		
	^238^U	^232^Th	^40^K
C1	51 ± 4	24 ± 2	238 ± 19
S1	55 ± 4	27 ± 2	238 ± 19
K1	59 ± 4	27 ± 2	238 ± 15
G1	55 ± 3	24 ± 2	223 ± 11
C2	55 ± 4	27 ± 2	242 ± 19
S2	59 ± 5	24 ± 2	242 ± 19
K2	55 ± 2	27 ± 3	238 ± 13
G2	55 ± 2	27 ± 2	219 ± 12
C3	59 ± 5	32 ± 3	247 ± 20
S3	63 ± 6	24 ± 2	257 ± 21
K3	55 ± 1	27 ± 4	242 ± 14
G3	51 ± 2	29 ± 5	242 ± 15
C4	55 ± 5	27 ± 2	238 ± 19
S4	63 ± 6	29 ± 2	257 ± 21
K4	59 ± 3	29 ± 4	252 ± 15
G4	55 ± 1	27 ± 2	238 ± 13
C5	47 ± 4	29 ± 2	247 ± 20
S5	59 ± 5	29 ± 2	247 ± 20
K5	55 ± 2	29 ± 3	238 ± 15
G5	55 ± 1	29 ± 4	238 ± 14
C6	59 ± 5	27 ± 2	247 ± 20
S6	55 ± 4	29 ± 2	252 ± 20
K6	51 ± 2	27 ± 2	242 ± 14
G6	51 ± 1	27 ± 2	238 ± 15
C7	55 ± 4	29 ± 2	238 ± 19
S7	55 ± 4	27 ± 2	242 ± 19
K7	55 ± 1	24 ± 1	238 ± 13
G7	59 ± 2	29 ± 3	247 ± 17
Average	56 ± 3	27 ± 2	242 ± 17
World average	33	45	420

**Table 2:** Elemental compositions and density of the samples.

Sample	Concentration by weight (%)											Density (g cm^−3^)
	Na	Mg	Al	Si	K	Ca	Ti	Mn	Fe	Zn	Ba	
Clay	11.25	0.63	16.37	36.83	12.91	BDL	2.12	0.10	19.09	0.05	0.68	1.62
Sand	4.73	0.40	13.34	49.36	12.88	BDL	1.31	0.05	16.48	0.01	1.44	1.66
Kaolin	1.56	1.65	22.25	55.03	4.28	11.86	0.55	0.08	2.66	BDL	0.08	1.94
Gypsum	0.03	0.33	0.14	0.34	0.04	96.46	BDL	0.01	2.65	BDL	BDL	2.28

From Table 2, it can be seen that high mean concentrations of Si and Al were found to be in kaolin with respective values of 55.03% and 22.25%, while that of K and Fe were found to be in clay with respective values of 12.91% and 19.09%, and that of Ba with an average value of 1.44% was found to be in Sand. High mean concentration of Ca was found to be in gypsum with a value of 96.46% which is in line with the fact that gypsum is known to be rich in calcium and informs its utilization as an essential component of cement^32^.

Similarly, silicon content was almost the same in all of the kaolin, sand, and clay samples, regardless of where they came from. This is not strange as it is well known that silicon, which is essentially abundant on the outermost layer of the earth's crust, is found in significant quantities in clay, sand, and kaolin^32^. On the other hand, elements like Mg, K, Ti, Mn, Zn, and Na, varied greatly in concentrations depending on the samples.

### Gamma ray shielding parameters results (GSP)

#### Gamma photon attenuation

**Figure 2:**
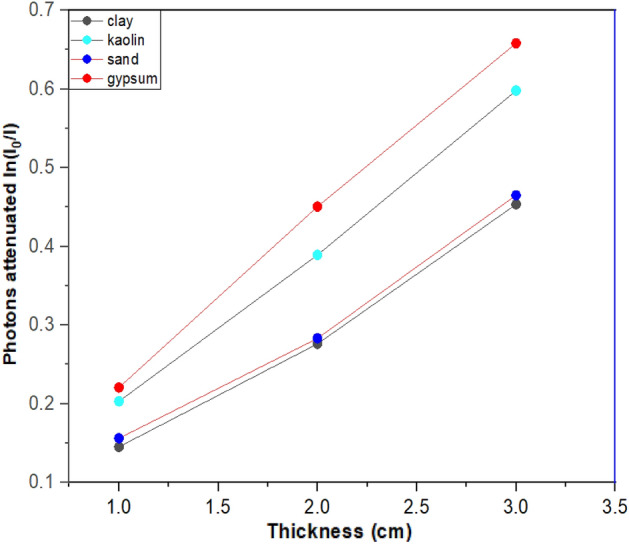
Attenuation of gamma rays against the ^137^Cs energy source as a function of sample thickness.

**Figure 3:**
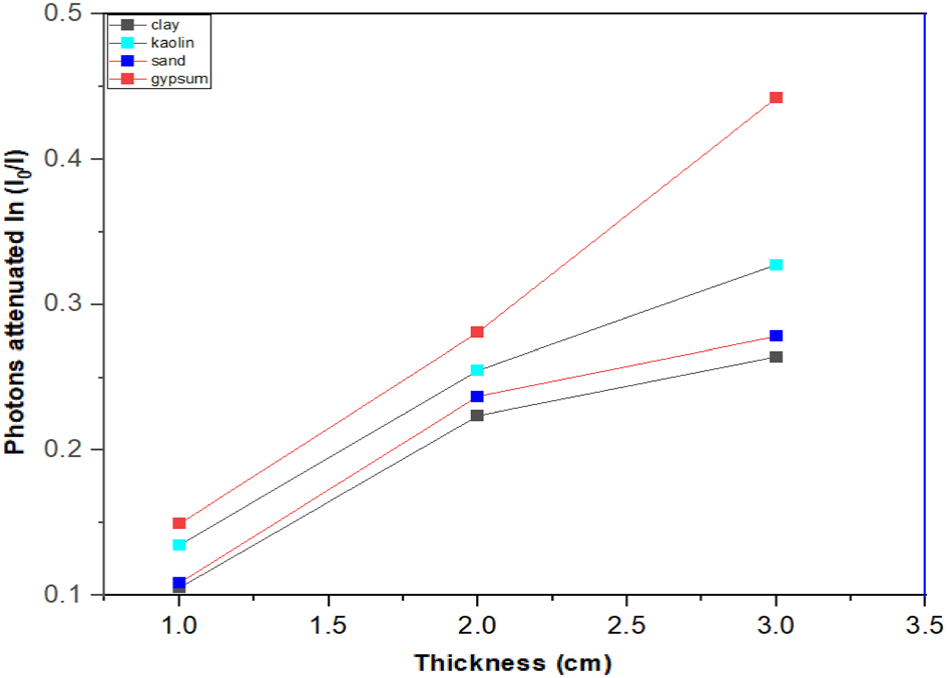
Attenuation of gamma rays against the ^60^Co energy source as a function of sample thickness.

Figures 2 and 3 presents the attenuation of photon beams from gamma radiation sources of ^137^Cs and ^60^Co peaks at various sample thicknesses. The results indicates that all the samples showed a rising behaviour as the thickness increased from 1 to 3 cm. The materials (gypsum, kaolin, and clay) were observed to respectively attenuates 63.5%, 61.5%, 58.4%, and 44.2% of gamma-ray photons at a thickness of 3 cm for a gamma energy of 0.662 MeV. This was also the case for gamma energy of 1.332 MeV, in which photons were attenuated by 40.6%, 32.9%, 30.6%, and 27.3% respectively. However, gypsum was observed to be the most effective material for significantly attenuating the photon beams followed by kaolin and clay samples. In addition to having a greater concentration of Ca and Fe than any other sample, gypsum was found to have a higher density than the other materials, clay and kaolin, respectively, which contributed to its superior performance. The general behaviour of all samples demonstrates a linear relationship between the sample thickness and the measured photon beam attenuation.

#### Mass attenuation coefficient (MAC) and linear attenuation coefficient (LAC)

**Figure 4:**
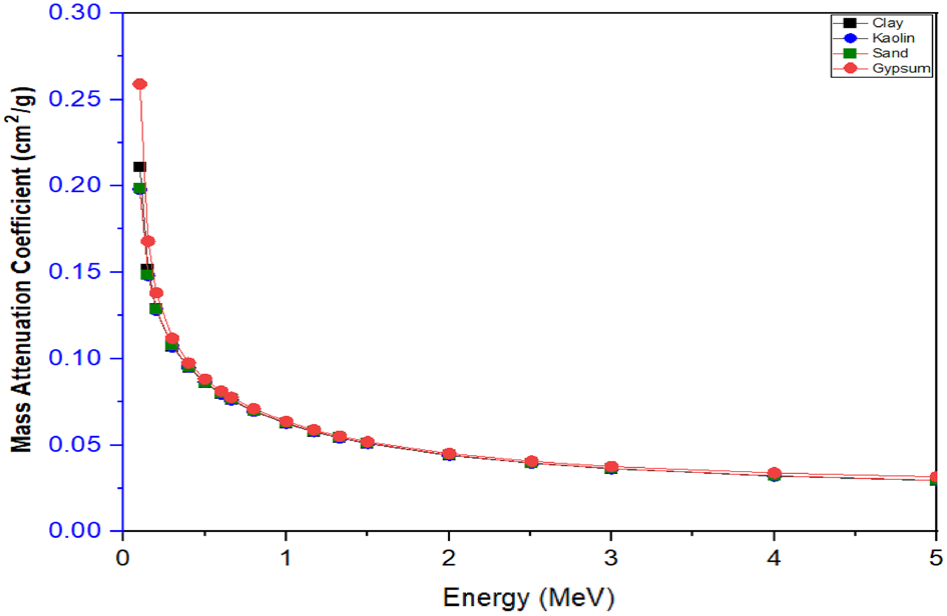
Mass attenuation coefficient as a function of photons energy of the samples.

**Figure 5:**
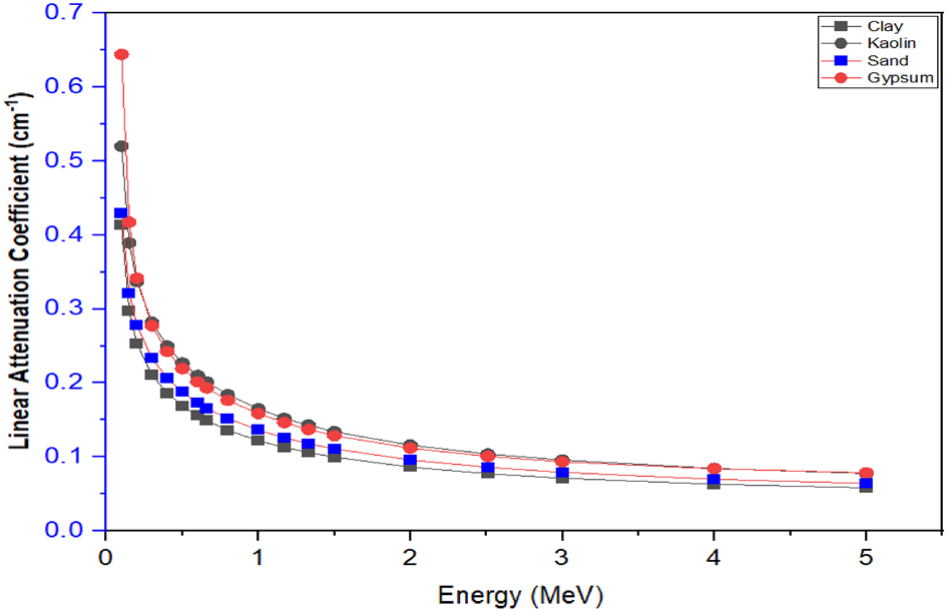
Linear attenuation coefficient as a function of photons energy of the samples.

The Mass Attenuation Coefficient (MAC) and Linear Attenuation Coefficients (LAC), were experimentally determined and compared with the theoretically computed results obtained using PY-MLBUF computer code of gamma energy ranges of (0.1–5.0) MeV^18^ and presented in Figs. 4 and 5, and Tables 3 and 4, respectively as a function of photon energy. It was observed that MAC and LAC decreases as photon energy increases. Similarly, as the photon energy increases from 0.1 MeV to about 0.8 MeV, MAC and LAC approaches comparable values for all the samples. Furthermore, at very low energies, MAC and LAC were observed to sharply drop as energy increases, which was attributed to the high inverse correlation between the photoelectric effect and photon energy in agreement with findings of Singh et al.^33^.

**Table 3:** Experimental and theoretical mass attenuation coefficient of the samples.

Mass attenuation coefficient (MAC) cm^2^/g						
Sample	0.662 MeV		1.173 MeV		1.332 MeV	
	Experimental	Py-MLBUF	Experimental	Py-MLBUF	Experimental	Py-MLBUF
Clay	0.0767 $\pm$ 0.0016	0.0760	0.0584 $\pm$ 0.0018	0.0576	0.0547 $\pm$ 0.0020	0.0541
Sand	0.0771 $\pm$ 0.0019	0.0765	0.0589 $\pm$ 0.0013	0.0581	0.0550 $\pm$ 0.0022	0.0545
Kaolin	0.0773 $\pm$ 0.0012	0.0763	0.0587 $\pm$ 0.0016	0.0579	0.0557 $\pm$ 0.0018	0.0543
Gypsum	0.0789 $\pm$ 0.0017	0.0778	0.0612 $\pm$ 0.0019	0.0589	0.0569 $\pm$ 0.0021	0.0552

**Table 4:** Experimental and theoretical linear attenuation coefficient of the samples.

Linear attenuation coefficient (LAC) (cm^−1^)						
Sample	0.662 MeV		1.173 MeV		1.332 MeV	
	Experimental	Py-MLBUF	Experimental	Py-MLBUF	Experimental	Py-MLBUF
Clay	0.152 $\pm$ 0.0019	0.149	0.121 $\pm$ 0.0018	0.113	0.109 $\pm$ 0.0020	0.106
Sand	0.184 $\pm$ 0.0016	0.178	0.147 $\pm$ 0.0013	0.135	0.131 $\pm$ 0.0022	0.126
Kaolin	0.209 $\pm$ 0.0013	0.201	0.159 $\pm$ 0.0016	0.152	0.148 $\pm$ 0.0018	0.143
Gypsum	0.228 $\pm$ 0.0018	0.207	0.166 $\pm$ 0.0019	0.157	0.153 $\pm$ 0.0021	0.147

It was further observed from Figs. 4 and 5 that the gypsum MAC and LAC values are higher than that of all the samples analyzed in the energy range, this is because MAC and LAC values are influenced by photon energy, density and the atomic number of the materials that are capable of changing photon attenuation in the samples in line with the work of Simon et al.^10^. However, the high values of MAC and LAC in gypsum could be due to the high content of Fe and Ca, and the high density of the sample compared to others.

Furthermore, Tables 3 and 4 presents a comparison of MAC and LAC results determined experimentally and theoretically using Py-MLBUF.

It can be observed from the tables that the results of the two methods are in good agreements with each other.

#### Half-value layer (HVL) and tenth value layer (TVL)

**Figure 6:**
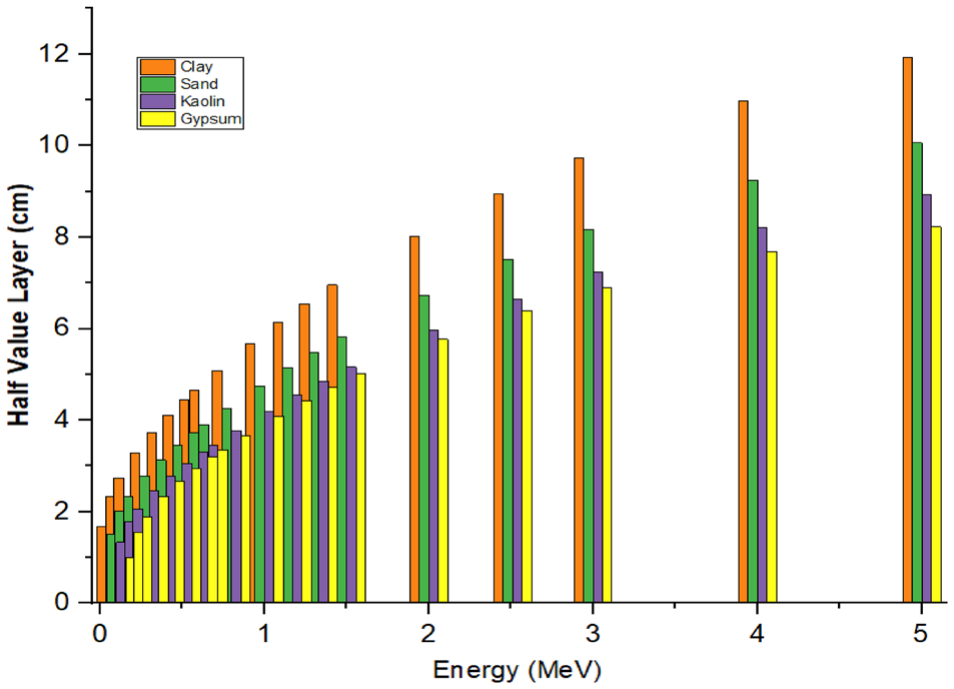
Half-value layer as a function of photons energy of the samples.

**Figure 7:**
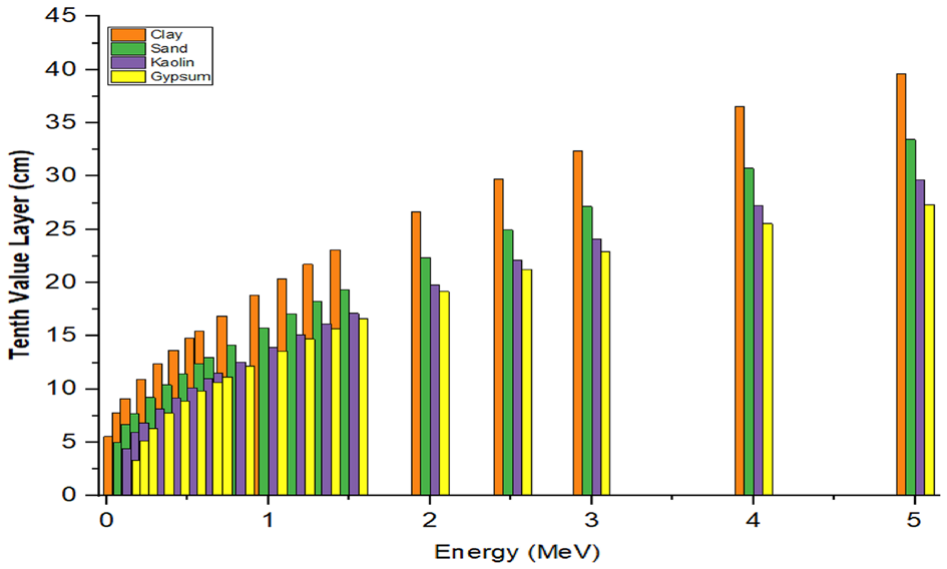
Tenth-value layer as a function of photons energy of the sample.

The HVL and TVL as a function of photon energy are presented in Figs. 6 and 7.

The figures showed that the HVL and TVL results increases with increment in incident photon energy. It can be observed that at very low energy region (< 0.8 MeV), the HVL and TVL values were almost the same for all the samples, due to the similar chemical contents of all the materials which agrees with the findings of Al-Jaffs^34^. It was also observed that the higher values of HVL and TVL occur as the energy increase reaches its maximum value at the highest energy region. Furthermore, it could be seen from the figures that as energy increases, the HVL and TVL results approach identical values for all the samples at gamma energy region of (0.1–0.8) MeV. However, as the photon energy increases above 2.0 MeV, a little increment in HVL and TVL values was observed and this behaviour could be due to the high Z-element dependency and the dominance of pair production at higher energy region as earlier stated by Al-Buriahi et al.^30^. It was also observed that all the samples have virtually same value at a specified gamma energy and this can be attributed to the same chemical compositions of the elements with high atomic number in the samples. Materials with low HVL and TVL were found to give better radiation shielding effectiveness due to low thickness of these materials which agreed with the work of Salisu et al.^35^. It was observed that HVL and TVL values are influenced by chemical contents, photon energy and density of the materials^36^. The HVL and TVL results for gypsum are low in comparison with other materials and this notified the highest density property in gypsum over other materials used in this work.

Tables 5 and 6 presents a comparison of HVL and TVL values determined experimentally and theoretically, in which a good agreement in the results of the two methods was observed.

**Table 5:** Experimental and theoretical half-value layer of the samples.

Half-value layer (HVL) (cm)						
Sample	0.662 MeV		1.173 MeV		1.332 MeV	
	Experimental	Py-MLBUF	Experimental	Py-MLBUF	Experimental	Py-MLBUF
Clay	4.8523 $\pm$ 0.0020	4.6544	6.3021 $\pm$ 0.0018	6.1356	6.6109 $\pm$ 0.0018	6.5426
Sand	4.1841 $\pm$ 0.0013	3.9037	5.3473 $\pm$ 0.0013	5.1432	5.5131 $\pm$ 0.0022	5.4823
Kaolin	3.6090 $\pm$ 0.0019	3.4552	4.6659 $\pm$ 0.0016	4.5520	4.9948 $\pm$ 0.0020	4.8534
Gypsum	3.4028 $\pm$ 0.0012	3.3507	4.5166 $\pm$ 0.0019	4.4247	4.8753 $\pm$ 0.0013	4.7207

**Table 6:** Experimental and theoretical tenth-value layer of the samples.

Tenth-value layer (TVL) (cm)						
Sample	0.662 MeV		1.173 MeV		1.332 MeV	
	Experimental	Py-MLBUF	Experimental	Py-MLBUF	Experimental	Py-MLBUF
Clay	15.5152 $\pm$ 0.0019	15.4617	20.7121 $\pm$ 0.0018	20.3822	21.9809 $\pm$ 0.0020	21.7340
Sand	13.0184 $\pm$ 0.0016	12.9677	17.8347 $\pm$ 0.0013	17.0853	18.7131 $\pm$ 0.0022	18.2118
Kaolin	11.9209 $\pm$ 0.0013	11.4780	15.6759 $\pm$ 0.0016	15.1214	16.8748 $\pm$ 0.0018	16.1227
Gypsum	11.5280 $\pm$ 0.0018	11.1307	14.8660 $\pm$ 0.0019	14.6984	15.9653 $\pm$ 0.0021	15.6819

#### Effective atomic number (Z_eff_)

**Figure 8:**
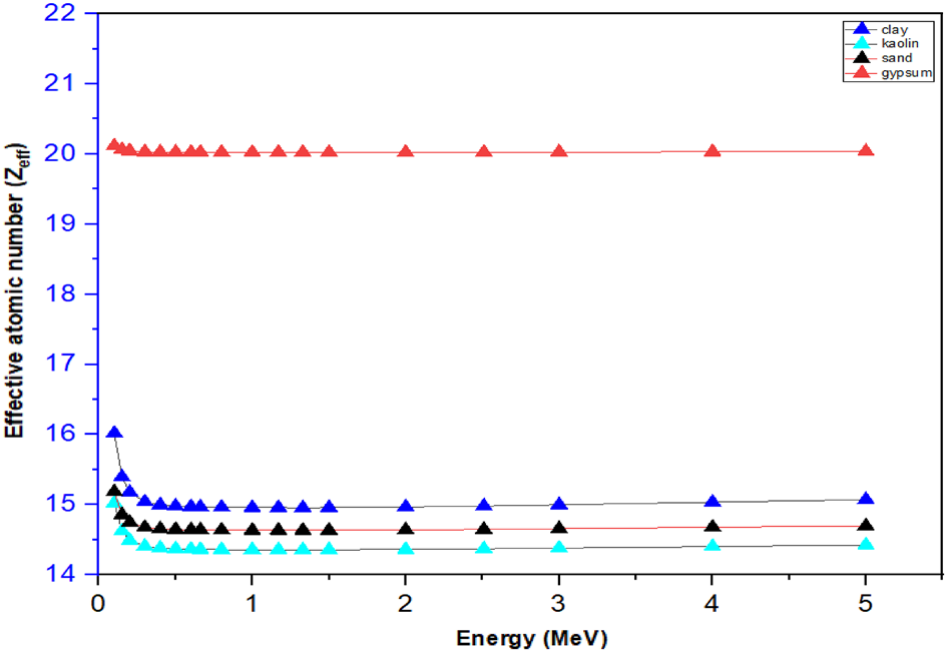
Effective atomic number (Z_eff_) as a function of photons energy of the samples.

Figure 8 present a plot of the effective atomic number *Z*_*eff*_ of the four samples against photon energy obtained using the Py-MLBUF program. It was observed that the values of *Z*_*eff*_ for all samples were higher at the lower energy region, this is primarily due to the photoelectric interaction which directly depends on the atomic number, Z^4^, and the photon energy as, E^−3.5^, for any absorber^37^.

At the intermediate photon energy, the *Z*_*eff*_ decreases slowly with the increase of incident photon energy and becomes almost independent of the photon energy for all the samples which agrees with the work of Akman et al.^38^. As the photon energy increases above 3.0 MeV, the value of *Z*_*eff*_ showed a little increment and this behaviour could be due to the high Z-element dependency and the dominance of pair production at higher energy region. At low energy region, the highest value of *Z*_*eff*_ was observed for gypsum and the minimum value was observed for clay sample, this indicated that *Z*_*eff*_ is linearly related to the Z-elements in the sample as earlier reported by Akman et al.^38^. Generally, it was observed that the *Z*_*eff*_ values of the samples decreased with increasing incident photon energies.

## Conclusion

Gamma-ray shielding parameters of locally used building materials (clay, sand, kaolin, and gypsum) in the Northwestern Nigeria were determined experimentally using ^60^Co and ^137^Cs gamma radiation standard point sources of photon peaks (0.662 and 1.133, 1.173) MeV and theoretically using Py-MLBUF software. The results showed that MAC and LAC values of the samples decrease with an increase in photon energy while HVL and TVL values increases. The experimental data of all the parameters were found to be in good agreement with the theoretical results obtained using Py-MLBUF. The studied samples (gypsum, kaolin, sand, and clay) were found to attenuate 63.5%, 61.5%, 58.4%, and 44.2% of gamma-ray photons with an energy of 0.662 MeV at a thickness of 3 cm, and 40.6%, 32.9%, 30.6%, and 27.3% of gamma energy of 1.332 MeV respectively. However, gypsum was found to have the best shielding behaviours and this is attributed to its high density (2.28 g cm^−3^) determined compared with the rest of the samples. The locally used building materials from the Northwestern Nigeria particularly gypsum can be used in shielding design and constructions of storage facility for X-ray and gamma ray sources and other low energies radioactive materials.

## Data Availability

All relevant datasets used in research work has been included in this paper as part of results.
